# Supplementary material for: Bevacizumab Treatment for Metastatic Colorectal Cancer in Real-World Clinical Practice
Source: Medicina (Kaunas). 2023 Feb 13;59(2):350. doi: 10.3390/medicina59020350 (PMC9963555; doi:10.3390/medicina59020350)
Supplement: Supplementary file 1 [file medicina-59-00350-s001.zip › Supplementary Material Table S1.pdf]

Suplementary Table S1. Metastatic disease characteristics

	Whole group (N=554)	First line (N=392)	Second line (N=162)	*p*-value
**Number of metastatic sites**				0.3350[2]
1	47 (8.5%)	33 (8.4%)	14 (8.6%)	
2	353 (63.7%)	243 (62%)	110 (67.9%)	
>2	154 (27.8%)	116 (29.6%)	38 (23.5%)	
**Metastases type**				0.2283[1]
Synchronous	380 (68.6%)	275 (70.2%)	105 (64.8%)	
Metachronous	174 (31.4%	117 (29.8%)	57 (35.2%)	
**Liver metastases**				>0.99[1]
Yes	431 (77.8%)	305 (77.8%)	126 (77.8%)	
No	123 (22.2%)	87 (22.2%)	36 (22.2%)	
**Bone metastases**				>0.99[1]
Yes	13 (2.3%)	9 (2.3%)	4 (2.5%)	
No	541 (97.7%)	383 (97.7%)	158 (97.5	
**Lung metastases**				0.9194[1]
Yes	169 (30.5%)	119 (30.4%)	50 (30.9%)	
No	385 (69.5%)	273 (69.6%)	112 (69.1%)	
**Peritoneal metastases**				>0.99[1]
Yes	120 (21.7%)	85 (21.7%)	35 (21.6%)	
No	434 (78.3%)	307 (78.3%)	127 (78.4%)	
**Brain metastases**				0.0250[1,*]
Yes	13 (2.3%)	5 (1.3%)	8 (4.9%)	
No	541 (97.7%)	387 (98.7%)	154 (95.1%)	
**Adrenal metastases**				0.4293[1]
Yes	18 (3.2%)	11 (2.8%)	7 (4.3%)	
No	536 (96.8%)	381 (97.2%)	155 (95.7%)	
**Abdominal Lymph nodes metastases**	58 (10.5%)	38 (9.7%)	20 (12.3%)	0.3623[1]
Yes	496 (89.5%)	354 (90.3%)	142 (87.7%)	
No				
**Splenic metastases**				0.0471[1,*]
Yes	12 (2.2%)	5 (1.3%))	7 (4.3%)	
No	542 (97.8%)	387 (98.7%)	155 (95.7%)	
**Cutaneous metastases**				0.0094[1,*]
Yes	8 (1.4%)	2 (0.5%)	6 (3.7%)	
No	546 (98.6%)	390 (99.5%)	156 (96.3%)	
**Pleural metastases**				>0.99[1]
Yes	2 (0.4%)	2 (0.5%)	0	
No	552 (99.6%)	390 (99.5%)	162 (100%)	
**Ovarian metastases**				>0.99[1]
Yes	12 (2.2%)	9 (2.3%)	3 (1.9%)	
No	542 (97.8%)	383 (97.7%)	159 (98.1%)	

[1], Fisher's exact test p-value; [2], Chi-square test p-value; *, significant difference.
