# Supplementary material for: Bevacizumab Treatment for Metastatic Colorectal Cancer in Real-World Clinical Practice
Source: Medicina (Kaunas). 2023 Feb 13;59(2):350. doi: 10.3390/medicina59020350 (PMC9963555; doi:10.3390/medicina59020350)
Supplement: Supplementary file 1 [file medicina-59-00350-s001.zip › Supplementary Material Table S2.pdf]

Table S2: Type of chemotherapy

Type of chemotherapy	Drug	Dose	Cycle length
FOLFOX4	Leucovorin<br>5-FU<br>Oxaliplatin<br>5-FU	400 mg/m2 bolus day 1+2<br>400 mg/m2 bolus day 1+2<br>85 mg/m2 over 2h<br>600 mg/m^2 over 22 hours days 1 and 2	14 days
mFOLFO6	Leucovorin<br>5-FU<br>Oxaliplatin<br>5-FU	400 mg/m^2 2h day 1<br>400 mg/m^2 bolus day 1<br>85 mg/m^2 2h day 1<br>2400 mg/m^2 continuous infusion 46 h	14 days
FOLFIRI	Leucovorin<br>5-FU<br>Irinotecan<br>5-FU	400 mg/m^2 2h day 1<br>400 mg/m^2 bolus day 1<br>180 mg/m^2 100 min day 1<br>2400 mg/m^2 continuous infusion 46 h	14 days
CAPOX	Oxaliplatin<br>Capecitabine	130 mg/m^2 2 h day 1<br>2000 mg/m^2 days 1-14	21 days
CAPIRI	Irinotecan<br>Capecitabine	250 mg/m^2 90 min day 1<br>2000 mg/m2 days 2-15	21 days
FOLFOX7	Leucovorin<br>Oxaliplatin<br>5-FU	400 mg/m^2 2h day 1<br>85 mg/m^2 2h day 1<br>2400 mg/m^2 continuous infusion 46 h	14 days
DeGramont	Leucovorin<br>5-FU<br>5-FU	200 mg/m^2 day 1 and day 2<br>400 mg/m^2 day 1 and day 2<br>600 mg/m^2 continuous infusion 22 h day 1 and day 2	14 days
mDeGramont	Leucovorin<br>5-FU<br>5-FU	400 mg/m^2 2h day 1<br>400 mg/m^2 2h day 1<br>2400 mg/m^2 continuous infusion 46 h	14 days
Capecitabine	Capecitabine	2500 mg/m2 z1-14	21 days
Nordic FLOX	5-FU<br>Leucovorin<br>Oxaliplatin	500 mg/m^2 bolus day 1 and day 2<br>60 mg/m2 bolus day 1 and day 2<br>85 mg/m^2 2h day 1	14 days
Nordic FLIRI	5-FU<br>Leucovorin<br>Irinotecan	500 mg/m^2 bolus day 1 and day 2<br>60 mg/m2 bolus day 1 and day 2<br>180 mg/m^2 100 min day 1	14 days
