# Supplementary material for: Bevacizumab Treatment for Metastatic Colorectal Cancer in Real-World Clinical Practice
Source: Medicina (Kaunas). 2023 Feb 13;59(2):350. doi: 10.3390/medicina59020350 (PMC9963555; doi:10.3390/medicina59020350)
Supplement: Supplementary file 1 [file medicina-59-00350-s001.zip › Supplementary Material Table S3.pdf]

Table S3: Prognostic factors for overall survival and progression free survival in patients with mCRC treated with bevacizumab

For patients with mCCR treated with bevacizumab in first line, the medians of PFS are the same across categories of chemotherapy regimen administered (irinotecan-based vs oxaliplatin-based vs fluoropyrimidine based chemotherapy, *p*-value=0.342). No differences were found in median of PFS according to the resection status of the primary tumor (Yes vs No, *p*-value = 0.198), the location of metastases (liver alone vs other, *p*-value = 0.69), number of metastatic sites (1 vs more, *p*-value = 0.081), type of metastases (metachronous vs synchronous, *p*-value = 0.166), RAS status (mutant vs non-mutant, *p*-value = 0.108).

Factor	Case (n,%)	Median PFS (months)	p-value	Median OS (months)	p-value
**Gender**			**0.638**		**0.782**
Male	234 (59.7%)	8.44		17.89	
Female	158 (40.3%)	8.42		17.56	
**Tumor location**			**0.220**		**0.003**
Left-sided	290 (74%)	8.67		19.23	
Right-sided	102 (26%)	7.86		14.37	
**Resection status of the primary tumor**			**0.198**		**0.005**
Yes	312	8.73		19.00	
No	77	7.27		13.71	
**Liver metastases**			**0.690**		**0.062**
Alone	305 (77.8%)	8.45		17.13	
Other	87 (22.2%)	8.42		22.52	
**No. of metastases**			**0.081**		**0.356**
1	33 (8.4%)	6.59		14.47	
≥2	359 (91.6%)	8.59		17.97	
**Type of metastases**			**0.166**		**0.010**
Metachronous	117 (29.8%)	9.24		22.03	
Synchronous	275 (70.2%)	8.15		16.60	
**RAS**			**0.108**		**0.070**
Mutant	133 (33.9%)	8.48		17.13	
Wild type	94 (24%)	9.24		20.27	
**Chemotherapy regimens**			**0.342**		**0.855**
Irinotecan-based	100 (25.5%)	9.16		19.82	
Oxaliplatin-based	274 (69.9%)	8.34		17.02	
Fluoropyrimidine-based	12 (3.1%)	9.21		15.01	
**Status**			**<0.0001**		**<0.0001**
Event	344 (87.8%)	8.03		15.79	
Censored	48 (12.2%)	16.32		31.14	
**Grading**			**0.875**		**0.550**
G1	88 (22.4%)	8.37		16.92	

G2	206 (52.6%)	9.14		18.76	
G3	33 (8.4%)	7.13		16.08	

For patients with mCCR treated with bevacizumab in second line:

Factor	Case (n, %)	Median PFS (months)	p-value	Median OS (months)	p-value
**Gender**			**0.546**		**0.717**
Male	105 (64.8%)	6.51		14.01	
Female	57 (35.2%)	6.61		12.10	
**Tumor location**			**0.611**		**0.190**
Left-sided	124 (76.5%)	6.64		14.32	
Right-sided	38 (23.5%)	6.45		11.53	
**Resection status of the primary tumor**			**0.049**		**0.009**
Yes	142	6.97		14.26	
No	19	4.80		7.96	
**Liver metastases**			**0.907**		**0.737**
Alone	126 (77.8%)	6.63		13.48	
Other	36 (22.2%)	6.38		13.08	
**No. of metastases**			**0.478**		**0.261**
1	14 (8.6%)	6.33		11.69	
≥2	148 (91.4%)	6.59		13.76	
**Type of metastases**			**0.824**		**0.595**
Metachronous	57 (35.2%)	6.58		13.94	
Synchronous	105 (64.8%)	6.61		12.66	
**RAS**			**0.006**		**<0.0001**
Mutant	33 (20.4%)	10.39		15.06	
Wild type	60 (37%)	7.25		20.32	
**Chemotherapy regimens**			**0.507**		**0.576**
Irinotecan-based	103 (63.6%)	7.30		12.89	
Oxaliplatin-based	35 (21.6%)	5.49		13.94	
Fluoropyrimidine-based	23 (14.2%)	6.87		12.10	
**Status**			**0.004**		**<0.0001**
Event	153 (94.4%)	6.31		12.59	
Censored	9 (5.6%)	21.70		56.45	
**Grading**			**0.827**		**0.503**
G1	52 (32.1%)	6.83		12.63	
G2	76 (46.9%)	6.64		14.08	
G3	14 (8.6%)	4.82		7.87	
